# Artificial Intelligence-Supported Performance-Based Assessment in Oral Radiology Education: A Constructive Alignment Perspective

**DOI:** 10.3390/diagnostics16162542

**Published:** 2026-08-12

**Authors:** Sinem Coşkun Albayrak, Fazıl Serdar Gürel, Gözde Kubat, Özlem Coşkun, Işıl İrem Budakoğlu, Kaan Orhan

**Affiliations:** 1 Department of Oral and Maxillofacial Radiology, Faculty of Dentistry, Lokman Hekim University, Ankara 06510, Turkey; sinem.coskun@lokmanhekim.edu.tr; 2 Department of Medical Education and Informatics, Başkent University, Ankara 06490, Turkey; fsgurel@gmail.com (F.S.G.); ozer.gozde@gmail.com (G.K.); 3 Department of Medical Education and Informatics, Gazi University, Ankara 06500, Turkey; drozlemcoskun@gmail.com; 4 Department of Medical Education and Informatics, Ankara Medipol University, Ankara 06050, Turkey; isiliremb@gmail.com; 5 Department of Oral and Maxillofacial Radiology, Faculty of Dentistry, Ankara University, Ankara 06500, Turkey; call53@yahoo.com

**Keywords:** artificial intelligence, assessment, automated pathology detection, constructive alignment, dental imaging, oral and maxillofacial radiology

## Abstract

**Background/Objectives:** This study aimed to examine the educational implications of an AI-supported performance-based assessment platform in oral radiology education within a constructively aligned framework. **Methods:** A total of 266 fourth-year dental students evaluated five panoramic radiographs using an AI-supported lesion detection platform with a predefined detection confidence threshold of 40%. Student performance was assessed using a confusion matrix framework, and precision and sensitivity values were calculated. Receiver operating characteristic (ROC) analysis was conducted to explore the relationship between region selection frequency and AI-supported diagnostic performance. Group comparisons were performed using the Mann–Whitney U test. **Results:** ROC analysis demonstrated a statistically significant but weak inverse association between region selection frequency and AI-supported diagnostic performance (AUC = 0.414, 95% CI: 0.342–0.486, *p* = 0.018). Students exceeding the cutoff of 8.5 marked regions demonstrated significantly lower performance scores than those at or below the cutoff (*p* < 0.001). No significant correlation was observed between AI-supported performance scores and traditional summative examination outcomes. **Conclusions:** AI-supported performance-based assessment demonstrated value in measuring applied diagnostic reasoning distinct from traditional written examinations. Within a constructively aligned framework, such tools may serve as complementary strategies for competency-oriented assessment in dental education.

## 1. Introduction

The purpose of undergraduate dental education is to develop competent dental professionals who possess the necessary knowledge, skills, and attitudes to meet society’s healthcare needs. Clinical competence is not limited to having clinical skills; it is a much more complex concept that emerges as a result of the integration of knowledge and performance and includes many characteristics, such as the use of knowledge, correct behavior and professionalism [1]. Effective radiology education is crucial to prevent missed diagnoses during image interpretation.

The use of artificial intelligence (AI) in dentistry has undergone significant growth and advancement in the last 20 years, both in terms of research and clinical trends. Successful AI radiological applications currently in use include anomaly identification, anatomic segmentation, picture quality assessment, natural language processing, and worklist improvement [2,3,4]. However, the integration of AI into dental education and practice is not without significant risks. Recent reports have identified AI-enabled health technologies as a top health technology hazard, citing risks of false or misleading results (“hallucinations”), algorithmic bias, and performance variability across patient populations [5]. In radiology education specifically, concerns regarding over-reliance on AI, automation bias, and the potential for “de-skilling” among trainees have been raised [6]. Clinicians may over-rely on AI-generated outputs and neglect their own clinical judgment, particularly when encountering atypical cases not adequately represented in training datasets [7,8]. These diagnostic hazards underscore the need for balanced, critical perspectives when integrating AI into educational curricula.

In higher education, AI has been applied to teaching and assessment in a variety of ways, including automated essay scoring, automated formative assessment, teaching evaluation, and individualized material delivery [9,10]. Various traditional assessment tools and evaluation methods are used in health education [11]. Many traditional assessment tools, including multiple-choice examinations and structured oral examinations, predominantly measure declarative knowledge and theoretical understanding [12]. While efficient for evaluating factual recall, these methods offer limited insight into students’ ability to apply knowledge in clinically authentic contexts, such as radiographic interpretation and lesion detection [13]. This misalignment between assessment format and intended learning outcomes may compromise the validity of competency-based evaluation frameworks [14].

AI-supported systems present an opportunity to address this gap by enabling performance-based assessment that mirrors real-world diagnostic tasks. AI platforms can provide standardized, objective, and immediate feedback on radiographic interpretation skills, thereby supporting the development of higher-order cognitive competencies [15]. However, the reliability of such assessments depends on expert validation of AI outputs, transparent scoring algorithms, and careful attention to the potential for automation bias among learners [16,17].

The theoretical foundation of this study is grounded in two established competency frameworks. Bloom’s revised taxonomy [18] classifies cognitive competencies along a hierarchy from remembering to creating; radiographic interpretation and differential diagnosis represent higher-order skills at the “analyze” and “evaluate” levels. Miller’s pyramid [19] distinguishes four levels of clinical competence: “Knows,” “Knows How,” “Shows How,” and “Does.” Traditional written examinations primarily assess the “Knows” and “Knows How” levels, whereas the AI-supported platform used in this study required students to actively localize lesions, delineate boundaries, and formulate diagnostic decisions without predefined options—thereby targeting the “Shows How” level of competence. This constructively aligned framework ensured that the assessment format was matched to the intended learning outcomes of applied diagnostic reasoning. The aim of this study was to examine whether an AI-supported performance-based assessment tool evaluates diagnostic competencies distinct from traditional written examinations within a constructively aligned oral radiology curriculum. Specifically, we sought to (i) characterize student performance on an AI-supported lesion detection task, (ii) explore the relationship between marking behavior and diagnostic accuracy, and (iii) compare AI-supported performance scores with traditional summative examination outcomes.

## 2. Materials and Methods

The study involved 266 out of a total of 280 fourth-year undergraduate dental students at the Faculty of Dentistry of Lokman Hekim University. The students were informed that they could withdraw from the study at any time. To prevent deprivation, the application was made available to all volunteer and non-volunteer students for 1 week, after which it was announced. Only data from students who signed the voluntary consent form were included in the study.

CranioCatch (Eskişehir, Türkiye) is a deep learning-based, CE-marked AI lesion detection system commercially available for clinical use in dental radiology. The platform utilizes convolutional neural networks (CNNs) trained on a multi-institutional dataset of panoramic radiographs, with reported sensitivity and specificity values exceeding 85% for common maxillofacial pathologies in prior validation studies. The system supports automated detection and classification of various maxillofacial lesions, including cysts, tumors, and fibro-osseous lesions, and is implemented as an educational assessment tool in the present study.

All AI-generated lesion annotations were independently reviewed and validated by two board-certified oral and maxillofacial radiologists (K.O. and S.C.A.) prior to student assessment. Cases with discordant AI annotations were discussed until consensus was reached, ensuring that the reference standard used for student grading was expert-verified rather than purely algorithmic.

Prior to the assessment session, all participants received standardized training on the use of the platform. The current curriculum of the fourth year includes panoramic radiographic imaging techniques and an analytical (systematic) strategy, which is one of the basic diagnostic methods and imaging features of maxillofacial lesions.

Five different panoramic radiographs containing lesions of different diseases that had not been previously examined were used in the study. Students were asked to detect possible pathological lesions in these radiographs and diagnose them with 40% threshold.

The 40% detection confidence threshold was selected based on the platform’s default educational setting, which was designed to balance lesion detection sensitivity with specificity in a training context. This threshold was chosen to ensure that the majority of clinically relevant lesions would be flagged by the AI, thereby providing students with a comprehensive reference standard while minimizing excessive false-positive annotations that could lead to alert fatigue.

Student performance was evaluated using a confusion-matrix-based framework comparing student-selected regions with expert-validated AI annotations. The following metrics were calculated:

Sensitivity (Recall) = TP/(TP + FN)

Precision = TP/(TP + FP)

where

- TP (True Positive): The lesion is correctly identified and diagnosed, with spatial overlap ≥50% between the student-marked region and the expert-validated annotation.
- FP (False Positive): One of three conditions: (i) the student’s diagnosis is correct but spatial overlap with the expert-validated annotation is <50%; (ii) spatial overlap is ≥50% but the diagnosis is incorrect; or (iii) the student marks and diagnoses a region where no true lesion exists on the radiograph.
- FN (False Negative): A true lesion exists on the radiograph but is neither marked nor diagnosed by the student.
- TN (True Negative): No lesion exists on the radiograph, and the student correctly does not mark or diagnose any region.

Composite Performance Score: For each radiograph, a composite score was calculated as the arithmetic mean of precision and sensitivity:

Composite Score = (Precision + Sensitivity)/2

The overall CranioCatch score for each student was calculated as the mean of composite scores across all five radiographs, yielding a value between 0 and 1. Precision and sensitivity values were displayed on a scoreboard for each lesion, and average precision and sensitivity values were calculated across all panoramic images.

The students were required to log into the AI education application using their unique usernames and passwords and start detection via computers or tablets. One hour was allowed for this examination. Real-time feedback to achieve learning outcomes at the end of the examination was given. For this purpose, students were allowed to learn about their grades, compare their own results with the AI results and see the results of the analysis at any time via the application.

With this AI-supported application, educators had the flexibility to intervene in the grading process, utilizing their judgment alongside the AI’s grading metrics. This dual approach allowed for a more nuanced evaluation of student performance.

In addition, a comparative evaluation was conducted to assess students’ overall annual performance and exam performance by comparing the effectiveness of the AI-supported application in assessment and evaluation with both the final exam scores and the end-of-year achievement. Representing traditional measurement methods, the faculty course board achievement scores were used. In this context, students’ end-of-year grades (calculated as a weighted average of midterm, practical, and final exam components) as well as their final exam scores were taken into account. Students who scored 60 or above on the final exam were classified as successful, whereas those who scored below 60 were classified as unsuccessful.

Researchers guaranteed that those who do not participate in the research also receive this training.

The effect of the AI-supported assessment and evaluation application was compared with the results obtained through traditional assessment and evaluation methods. In the traditional method, the end-of-year achievement scores were based on the questions designed to address the learning objectives within the scope of oral radiology.

### 2.1. Statistical Analyses

Within the scope of the study, statistical analysis of the data was carried out via the IBM Statistics SPSS 25 package program. As the data were not normally distributed, non-parametric statistical methods were applied, and descriptive statistics are presented as median values. In this study, a cutoff point was determined via the “sensitivity” and “1-specificity” values. In group comparisons, success scores were listed, and groups were created according to the cutoff point, in addition to the 27% and 50% groups. Receiver operating characteristic (ROC) curve analysis was conducted to examine whether region selection frequency (number of marked regions) could discriminate between higher and lower performers on the AI-supported task. Sensitivity and specificity were calculated across thresholds of region selection frequency, and a cutoff value was selected for exploratory group comparisons. Mann–Whitney U test statistics were used in the difference analyses carried out in line with the purpose of the study, and results are reported as median (interquartile range) for non-normally distributed data.

Correlation analyses were performed to examine the relationship between CranioCatch scores and students’ end-of-year achievement grades and final exam scores. As the variables were not normally distributed, relationships between continuous variables were evaluated using Spearman’s rank correlation coefficient. The association between CranioCatch scores and final exam success (≥60/<60) was examined using point-biserial correlation coefficient.

The obtained statistical analysis results were evaluated according to the 95% confidence level, and *p* values < 0.05 were considered statistically significant.

### 2.2. Use of Generative AI

During the preparation of this manuscript, ChatGPT (OpenAI, GPT-5.5) was used solely to improve the clarity, grammar, and academic writing of the text. The Generative AI tool was not used for study design, data collection, data analysis, data interpretation, statistical analyses, or figure generation. All scientific content was reviewed, verified, and approved by the authors, who take full responsibility for the final manuscript.

## 3. Results

Two hundred and sixty six (172 female, 94 male) undergraduate students participated in the study. Pathologies of the maxillofacial region were detected in 5 different panoramic images via AI application by all individuals.

**Figure 1:**
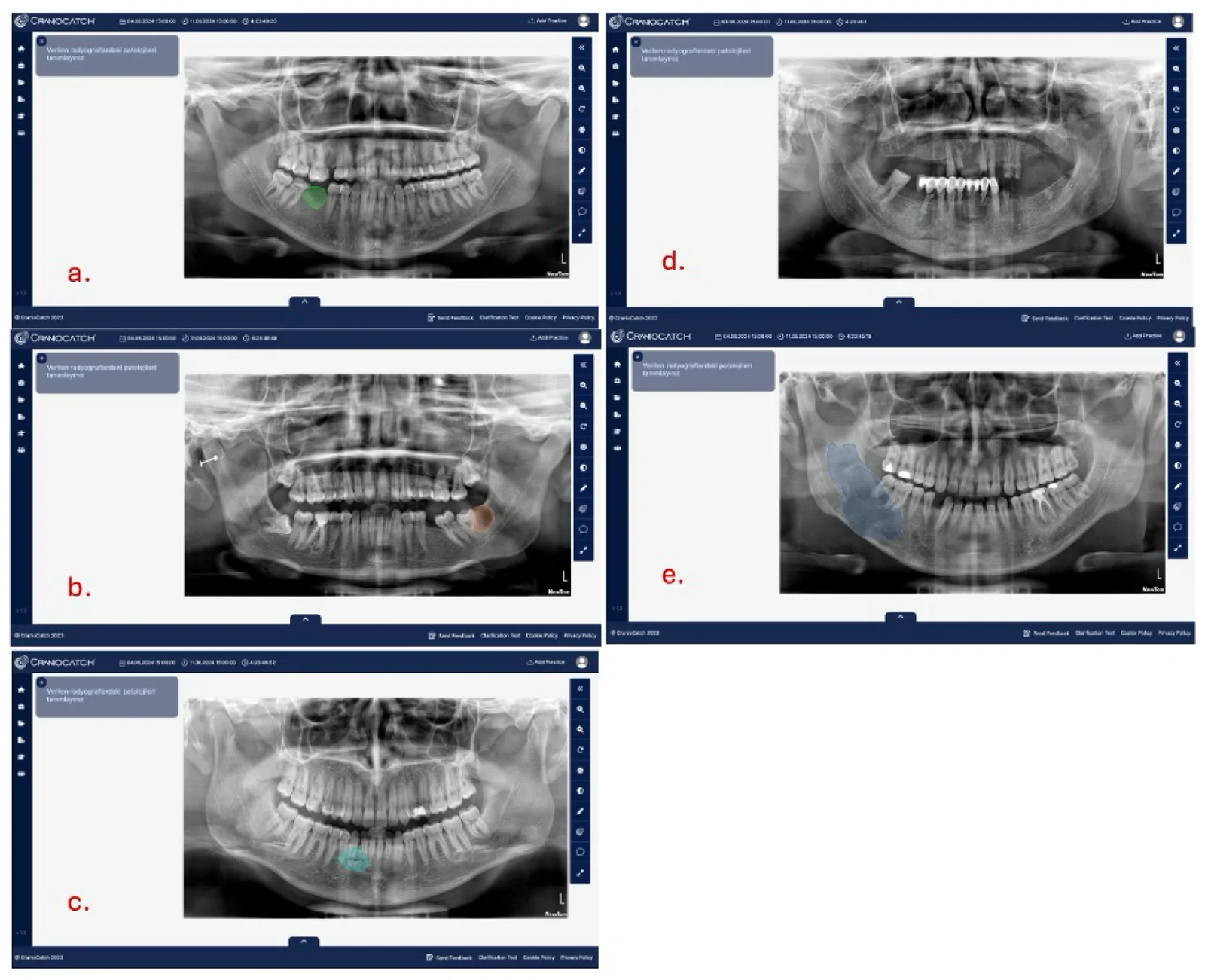
Maxillofacial pathologies detected by AI application: (**a**) fibrous dysplasia; (**b**) dentigerous cyst; (**c**) compound odontoma; (**d**) radicular cyst; (**e**) ameloblastoma.

Five panoramic radiographs containing pathologies of varying diagnostic complexity were selected. The lesions included: (a) fibrous dysplasia (right mandibular molar region)—a benign fibro-osseous lesion with characteristic ground-glass radiopacity and poorly defined margins, representing a moderately challenging diagnosis; (b) dentigerous cyst (left impacted mandibular third molar)—a common developmental cyst associated with an impacted tooth, considered a standard undergraduate-level case; (c) compound odontoma (left mandibular canine region)—a benign odontogenic tumor with multiple small tooth-like structures, representing an intermediate diagnostic challenge; (d) radicular cyst (periapical regions of teeth 12, 34, and 45)—the most common odontogenic cyst, typically taught as a foundational pathology in dental curricula; and (e) ameloblastoma (right mandibular molar and ramus region)—a benign but locally aggressive odontogenic tumor with multilocular radiolucency, considered an advanced diagnostic case (Figure 1). Collectively, these cases span a spectrum from common, curriculum-standard pathologies (dentigerous cyst, radicular cyst) to more complex, less frequently encountered lesions (fibrous dysplasia, ameloblastoma), thereby providing a balanced assessment of students’ diagnostic competencies.

**Figure 2:**
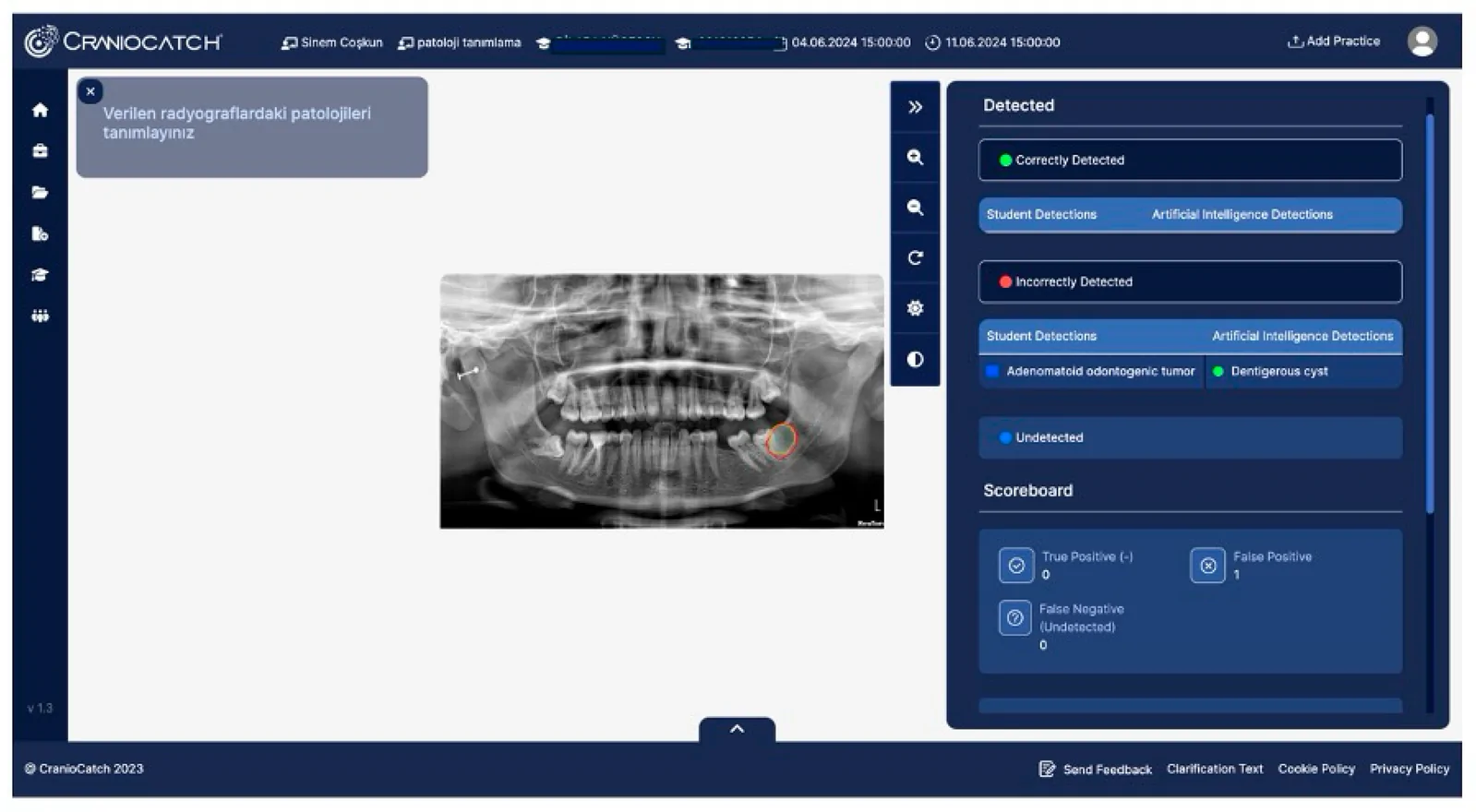
Comparison of student and application diagnoses and superimposed visual images of lesion locations.

Student responses were compared with AI annotations regarding lesion location and diagnosis. As illustrated in Figure 2, the diagnosis and location of the lesions were visualized by overlapping the student response and the AI response with different color codes.

**Figure 3:**
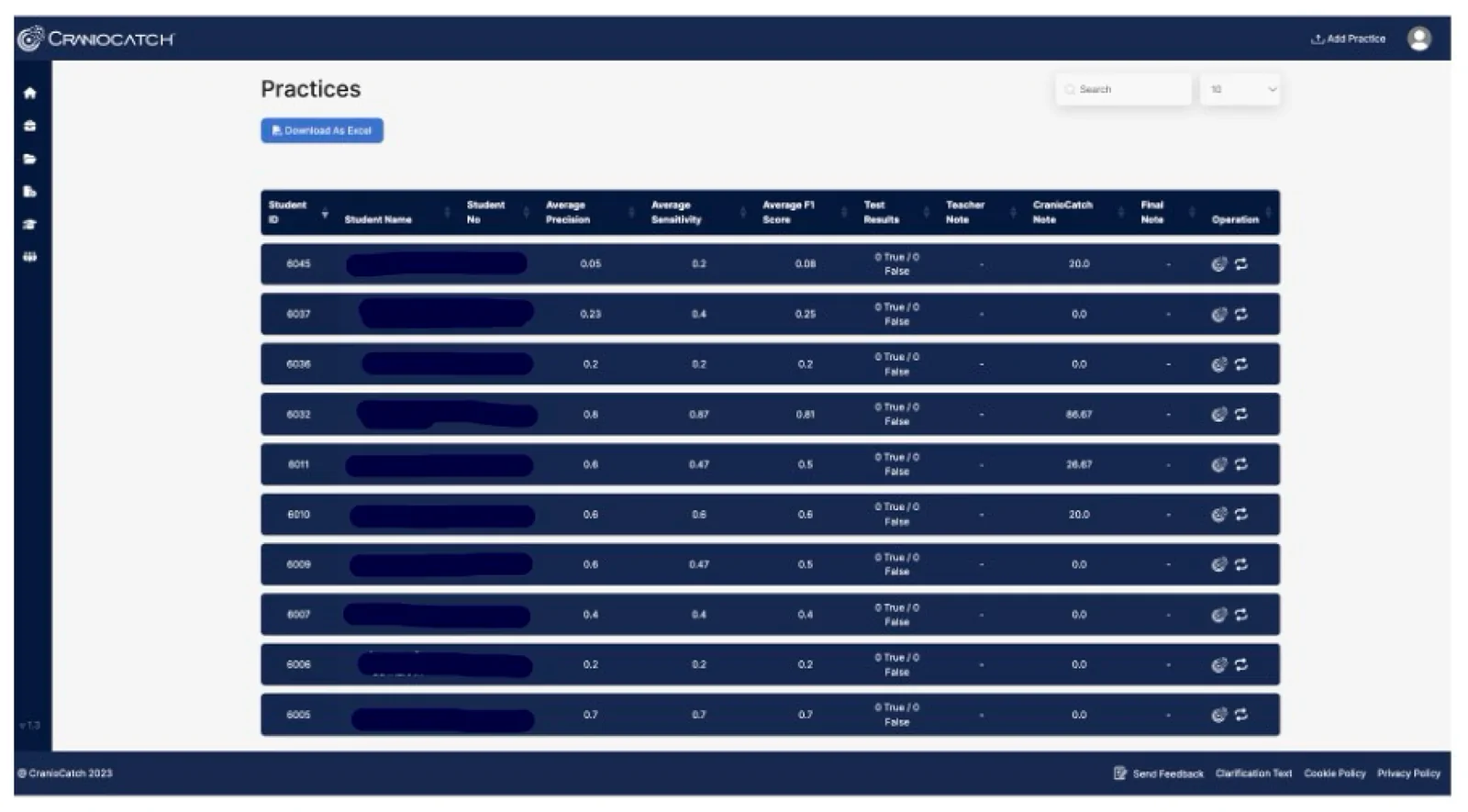
Average precision and sensitivity for each student in the virtual classroom.

As illustrated in Figure 3, grading was performed through the application according to the accuracy of lesion detection and diagnosis for each student. Immediate feedback was provided upon completion of the assessment, allowing students to review their scores and compare their responses with the expert-validated AI results.

To explore performance distribution patterns, comparisons were conducted between the upper and lower 27% and 50% groups based on region selection frequency. Specifically, within the 27% subgroup, students in the upper 27% marked a mean of 5.56 ± 1.58 regions, whereas students in the lower 27% marked 17.03 ± 5.55 regions. A statistically significant difference was observed between these groups (*p* < 0.001). Comparisons between the upper and lower 50% performance groups also revealed a statistically significant difference in region selection frequency (*p* < 0.001).

**Figure 4:**
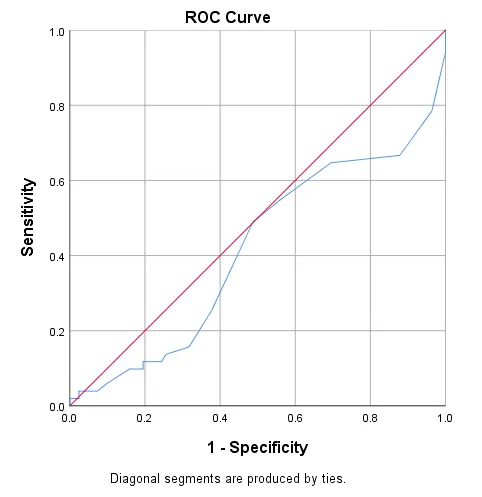
Receiver operating characteristic (ROC) curve illustrating the relationship between region selection frequency and AI-supported diagnostic performance.

Receiver operating characteristic (ROC) curve analysis identified an exploratory cutoff of 8.5 marked regions for subsequent group comparisons. ROC analysis yielded an AUC of 0.414 (95% CI: 0.342–0.486, *p* = 0.018), demonstrating a statistically significant but weak inverse discrimination, indicating that region selection frequency does not provide meaningful classification accuracy for AI-supported task performance (Figure 4).

Using this exploratory cutoff of 8.5 marked regions, sensitivity was 0.549 and specificity was 0.561, indicating limited classification accuracy (Table 1). Using the cutoff of 8.5 marked regions, students who exceeded this threshold achieved significantly lower AI-supported performance scores than those at or below the cutoff (*p* < 0.001).

No significant relationship was found between CranioCatch scores and students’ end-of-year achievement grades (ρ = 0.06, *p* = 0.328). Similarly, a weak positive correlation was observed between CranioCatch scores and final exam scores; however, this relationship did not reach statistical significance (ρ = 0.11, *p* = 0.082). Furthermore, point-biserial correlation analysis based on the classification of final exam performance as pass (≥60) or fail (<60) revealed no significant association with CranioCatch scores (r_pb = 0.08, *p* = 0.213). Overall, correlation analyses showed no statistically significant relationships between CranioCatch performance scores and traditional academic indicators (Table 2).

Spearman’s rank correlation coefficients (ρ) are reported for associations between continuous variables. Point-biserial correlation (r_pb) was used for the dichotomous variable final exam success (≥60). All *p* values are two-tailed. N = 266.

## 4. Discussion

From a constructive alignment perspective, discrepancies between assessment outcomes may arise when assessment formats target different competency domains [20]. In this study, radiographic interpretation and lesion detection were explicitly conceptualized as performance-based competencies aligned with the “Shows How” level of Miller’s pyramid [19] and the “analyze” and “evaluate” levels of Bloom’s revised taxonomy [18]. Traditional written examinations in dental education predominantly assess declarative knowledge and structured reasoning (“Knows” and “Knows How”), whereas the AI-supported platform required students to localize lesions, delineate boundaries, and formulate diagnostic decisions without predefined options, thereby engaging higher-order cognitive skills such as application, analysis, and evaluation [15,16,17].

Accordingly, the absence of a significant correlation between AI-supported and traditional assessment scores likely reflects differences in construct emphasis rather than methodological inadequacy. These domains should therefore be understood as distinct yet complementary dimensions of competence, as AI-supported performance scores may capture aspects of applied diagnostic reasoning not fully represented in traditional summative examinations.

By enabling lesion detection, boundary delineation, and independent diagnostic decision-making, AI-supported platforms facilitate authentic performance-based evaluation. The integration of immediate feedback further supports reflective learning processes, while standardized digital scoring may reduce variability associated with instructor-dependent assessment. Together, these features suggest that AI-supported tools can enhance the comprehensiveness and ecological validity of radiographic competency assessment within dental education.

The present findings should also be considered within the broader transition toward authentic and technology-enhanced assessment in health professions education. Traditional assessment approaches may inadequately capture the application of knowledge and higher-order competencies in clinically relevant contexts, whereas authentic assessment seeks to evaluate learners through tasks that more closely reflect real-world professional practice [21,22,23,24]. In this context, AI has increasingly been recognized not only as a subject that healthcare professionals need to understand, but also as a potential component of educational and assessment strategies [25]. This development is particularly relevant to radiology, where the recent literature has emphasized the growing need to integrate AI-related competencies into training curricula [26,27,28]. Evidence from structured educational initiatives further suggests that dedicated AI training can improve radiology trainees’ knowledge and understanding of AI applications [29]. The present study extends this literature by examining AI integration from a complementary perspective: rather than focusing solely on AI literacy or knowledge acquisition, the platform was incorporated into an assessment task requiring students to localize radiographic abnormalities, delineate lesion boundaries, and formulate diagnostic decisions. Accordingly, AI-supported assessment may provide an additional means of evaluating applied radiographic reasoning when implemented within a constructively aligned educational framework.

While AI-supported platforms offer significant educational benefits, their integration into assessment should not obscure the diagnostic and implementation risks associated with AI in clinical practice. AI systems may produce false or misleading outputs, exhibit variable performance across patient populations, and remain vulnerable to algorithmic bias, limited generalizability, and insufficient transparency in decision-making processes [5,7,8,30]. The recent literature has further emphasized that technical performance alone is insufficient for safe clinical adoption, as reproducibility, out-of-distribution generalization, regulatory considerations, and the continued need for clinician oversight remain important barriers to autonomous AI implementation [30]. In educational settings, these concerns are particularly relevant because excessive reliance on AI-generated outputs may contribute to automation bias and potentially interfere with the development of independent diagnostic reasoning among trainees [6]. Therefore, AI-supported assessment should be implemented as a supervised educational strategy rather than as a substitute for expert judgment, with transparent scoring procedures, expert-validated reference standards, and explicit emphasis on critical appraisal of AI outputs [7,8].

An additional noteworthy finding was the inverse association between marking frequency and diagnostic performance. With an AUC below 0.5 (AUC = 0.414), ROC analysis revealed a weak inverse discriminative pattern rather than meaningful classification accuracy, suggesting that region selection frequency alone cannot reliably differentiate higher from lower performers. Students who exceeded the exploratory cutoff of 8.5 marked regions demonstrated significantly lower AI-supported performance scores compared to those at or below the cutoff. This pattern may reflect a “shotgun” approach to lesion detection, where less confident students mark multiple regions in the hope of capturing true positives, thereby increasing false-positive rates and reducing precision.

The absence of a significant correlation between AI-supported and traditional assessment scores should be interpreted cautiously. While this discrepancy may reflect differences in construct emphasis—specifically, the distinction between declarative knowledge (assessed by written examinations) and applied diagnostic reasoning (assessed by the AI platform)—alternative explanations must also be considered. These include measurement error, the limited number of cases assessed, and potential variability in the AI platform’s discriminatory capacity.

We acknowledge that the flexibility afforded to educators to intervene in the grading process may introduce subjectivity and limit the objectivity of the assessment. Although this feature was designed to allow expert judgment to override potential AI errors, it also means that the final scores may reflect a combination of AI-generated metrics and instructor discretion. Future implementations should consider fully automated, algorithmic scoring to enhance objectivity, or alternatively, should systematically evaluate inter-rater reliability when instructor override is permitted.

This study has several limitations. The single-institution design and inclusion of only fourth-year students may restrict generalizability, and the relatively limited sample size of five radiographs further constrains broader extrapolation of the findings. The diagnostic complexity of the selected cases, while spanning a range from common to advanced pathologies, may not fully represent the spectrum of maxillofacial diseases encountered in clinical practice. In addition, the absence of longitudinal follow-up limits conclusions regarding sustained learning effects. Differences in construct emphasis between AI-supported and traditional assessments may also have influenced comparative outcomes. Finally, the platform’s native performance characteristics at the 40% confidence threshold setting may have influenced the sensitivity of the initial AI-generated annotations to subtle lesions. Although all AI-generated annotations were subsequently reviewed and verified by expert oral and maxillofacial radiologists, thereby reducing the potential influence of AI-related false-positive and false-negative outputs on the final reference standard, the possible effect of the selected threshold cannot be entirely excluded.

## 5. Conclusions

AI-supported assessment in oral radiology education demonstrated value in measuring diagnostic performance and applied clinical decision-making. Although no significant correlation was observed with traditional examination outcomes, this finding should be interpreted cautiously, as it may reflect differences in construct emphasis, measurement variability, or the limited scope of the assessment task. Within a competency-based and constructively aligned framework, AI-supported tools may serve as complementary—rather than substitutive—assessment strategies, offering authentic and clinically relevant evaluation of radiographic interpretation skills. However, the objectivity of such tools depends on transparent scoring algorithms and minimal instructor intervention. The integration of real-time feedback and digital performance analytics further supports reflective learning processes. For summative assessment contexts, careful case design—such as limiting the number of lesions per radiograph and ensuring balanced representation of common and complex pathologies—may enhance discriminatory capacity. Future research incorporating multi-institutional cohorts, longitudinal designs, fully automated scoring frameworks, and integrated assessment approaches is warranted to further clarify the role of AI-supported platforms in competency-oriented dental education.

## Figures and Tables

**Table 1:** Region selection frequency across stratified groups.

	Mean ± Standard Deviation	*p*
The top 27% of the performers	5.56 ± 1.58	*p* < 0.001 *
The bottom 27% of the performers	17.03 ± 5.55	
The top 50% of the performers	6.68 ± 1.76	*p* < 0.001 *
The bottom 50% of the performers	13.90 ± 5.32	
Less than the cutoff value of 8.5	6.39 ± 1.64	*p* < 0.001 *
Greater than the cutoff value of 8.5	13.40 ± 5.26	

* *p* < 0.05.

**Table 2:** Correlations between CranioCatch scores, end-year grades, and final exam scores.

	CranioCatch Score	End-Year Grade	Final Exam Score	Final Exam Success (≥60)
CranioCatch score	—	ρ = 0.06, *p* = 0.328	ρ = 0.11, *p* = 0.082	r_pb = 0.08, *p* = 0.213
End-year grade	ρ = 0.06, *p* = 0.328	—		
Final exam score	ρ = 0.11, *p* = 0.082		—	
Final exam success (≥60)	r_pb = 0.08, *p* = 0.213			—

## Data Availability

The datasets used and/or analyzed during the current study are available from the corresponding author on reasonable request.

## Abbreviations

The following abbreviations are used in this manuscript:

AI	Artificial Intelligence
AUC	Area Under the Curve
CNN	Convolutional Neural Network
FP	False Positive
FN	False Negative
ROC	Receiver Operating Characteristic
TN	True Negative
TP	True Positive
